# Metagenomic insights into the modulatory effects of kelp powder (*Thallus laminariae*)-Treated dairy milk on growth performances and physiological lipometabolic processes of kunming mice

**DOI:** 10.3389/fnut.2022.949809

**Published:** 2022-09-26

**Authors:** Fuguang Xue, Qingnan Mo, Pengyun Ma, Jian Zhang, Shuzhen Wang, Chuanxia Zheng, Yuqin Sun, Minze Liu, Zhengang Yang, Hao Bai

**Affiliations:** ^1^Nanchang Key Laboratory of Animal Health and Safety Production, Jiangxi Agricultural University, Nanchang, China; ^2^Joint International Research Laboratory of Agriculture and Agri-Product Safety, The Ministry of Education of China, Institutes of Agricultural Science and Technology Development, Yangzhou University, Yangzhou, China; ^3^Yangxin Yiliyuan Halal Meat Co. Ltd., Yangxin, China

**Keywords:** kelp-treated milk, mice, PUFAs, lipometabolic, gut microbiota

## Abstract

Kelp powder, supplemented with a dairy cow diet, effectively improved the milk polyunsaturated fatty acids (PUFAs) content. However, little information exists on the downstream effects of the kelp-treated milk on body health, gut microbiota, and nutrient metabolism. For this purpose, 48 3-week old Kunming (KM) male mice with an average body weight of 16.1 g ± 0.2 g were randomly divided into the control treatment (CON, fed with standard chow), the common milk supplement treatment (Milk), and the kelp powder-treated milk supplement treatment (KPM). The experiment lasted for 35 days, with a 7-day long adaptive period and a 28-day long main trial. Phenotypic parameters including growth performances and serum lipids-related parameters were first measured, and results indicated that Milk and KPM supplement significantly promoted the total body weight gain (*P* < 0.05), while significantly decreasing the feed conversion ratio compared with CON (*P* < 0.05). No significant differences were observed in the blood lipids content among all three treatments, however, the triglyceride content showed a decreasing trend after KPM supplement treatment. Further, activities of liver lipometabolic-related enzymes were investigated to determine the underlying factors that impacted physiological lipid metabolism. KPM treatment showed a significant reductive effect on the activity of lipogenesis-related enzymes, such as FAS and ACC, while a significant stimulative effect on the activity of lipolysis-related enzymes included the ATGL and CPT1 compared with CON (*P* < 0.05). Finally, gastrointestinal tract development and cecal microbiota community that correlated with body lipid degradation and absorption were measured to determine the underlying mechanism of KPM supplementation on physiological lipid metabolism. Results indicated that supplementation with KPM significantly enhanced cecal bacteria diversity which was reflected in the significant increase of Chao1 and ACE indexes. Besides, starch-degraded bacteria such as *Faecalibacterium, Ruminococcaceae*, and *Streptococcus* are significant decreased (*P* < 0.05), while cellulose-degraded bacteria including *Parabacteroides, Prevotella, Lactobacillus, Clostridium*, and *Bifidobacterium* are significantly increased (*P* < 0.05) after KPM supplement, which may further restrict the energy generation and therefore reduce the lipid deposition. In summary, kelp supplement helped increase the milk PUFAs content, enhance the bacterial diversity and relative abundances of probiotics, which finally modulated physiological lipid metabolism, and promote growth performances.

## Introduction

Dairy milk provided humans with broad essential nutrients (proteins and essential fatty acids) and bioactive ingredients (polyunsaturated fatty acids and lactoferrin), which functionally enhanced physiological immunity, proliferated intestinal flora, lowered serum cholesterol, alleviated antioxidation, and promoted the body's health (1–3). Milk fat content needs to be specially pointed out for not only the impacts on flavor (4) but also the potential health-promoting effects because of the content of essential fatty acids (EFAs), including the monounsaturated (MUFA) and the polyunsaturated fatty acids (PUFA, especially n-3 polyunsaturated fatty acids) (5–7).

Highly purified omega-3 PUFAs generally showed an effective regulatory effect on a certain number of genes and enzymes enriched in carbohydrate metabolism, energy provision, and body obesity (8). Increment in PUFA content also helps promoting the development of the intestinal tract and further significantly increasing nutrient utilization (9). Moreover, PUFAs functionally attenuated inflammatory bowel disease (IBD) through regulatory effects on gut bacterial diversity, which included the effective proliferation of probiotics, such as *Bifidobacterium* (10–12). However, PUFAs in mammals are less efficiently synthesized due to restrictions of certain essential factors including precursors preparation, fatty acids construction, α-linolenic acid (ALA, C18:3), and several elongations and desaturation chemical reactions (13). Fortunately, increasing potency of PUFA content could be achieved by dietary management such as supplementation with marine organisms.

Marine organisms, such as microalgae, seaweeds, and kelp have been proved of PUFAs abundance and could be further directly absorbed by the mammary gland (14, 15) or efficiently triggered the activity of *de novo* PUFAs synthesis, which finally significantly enhanced the milk PUFAs content (16). However, not much is known about the follow-up benefits of kelp-treated milk on gut microbiota, nutrient metabolism, and physiological body health. Therefore, in the present study, kelp-treated milk was supplemented into mice straw to investigate the regulatory effects on gut microbiota and physiological nutrient metabolism. We hypothesized that supplementary of proper amount of kelp-treated milk could work as PUFAs to help promote the growth performances through modulation of the physiological lipid metabolism and proliferation of the relative abundances of probiotics.

## Materials and methods

Animal care and procedures followed The Chinese Guidelines for Animal Welfare, which was approved by the Animal Care and Use Committee of Jiangxi Agricultural University, with the approval number JXAULL-20220226.

### Milk sources acquirement

The milk used in the present treatment was acquired from the mix of 12 Chinese Holstein lactating dairy cows [609 ± 17.9 kg BW; 170 ± 18 DIM, milk yield (25 ± 1.3 kg/day)]. Six of which received a 28-day long control diet (CON), while the rest were treated with kelp powder replacing diet (Kelp), respectively. Dietary formation and kelp supplementary methods were stated in our previous study (16). To be simply stated, the kelp powder (brown algae, purity 100%) was purchased from Xiamen Huison Biotech Co., Ltd, Xiamen, Fujian Province, China (http://www.chinahuison.com/). Kelp was used partly to replace corn silage and oat hay which resulted in kelp content coming to 5% [dry matter (DM) basis]. The diets were formulated according to NRC (2001) to meet or exceed the energy and protein requirements of Holstein dairy cows yielding 25 kg of milk/day with 3.5% milk fat and 3.0% true protein. Details of ingredient analysis and chemical composition of diets were shown in Supplementary Table 1.

Milk samples were acquired during the morning milking process at about 08:00 through milking facilities (90 Side-by-Side Parallel Stall Construction, Afimilk, Israel) every day. Milk from each treatment was mixed well, and stored in 100-mL vials in ice boxes before offering to mice. Milk quality of each treatment was measured on the last consecutive 3 days including milk protein, milk fat, somatic cell count (SCC), and colony forming unit (CFU). Milk quality measurement results are shown in Table 1.

**Table 1:** Measurement of the milk quality and milk fatty acids composition of both common milk and kelp-treated milk.

**Items**	**Milk**	**KPM**	***P*-value**
Milk fat (%)	3.61 ± 0.12	3.72 ± 0.08	0.012
Milk protein (%)	3.42 ± 0.02	3.39 ± 0.03	0.107
Milk dry matter content (%)	12.62 ± 0.13	12.67 ± 0.11	0.061
CFU (× 10^3^/mL)	2.40 ± 0.23	2.33 ± 0.13	0.722
SCC (× 10^3^/mL)	8.80 ± 0.64	9.20 ± 0.65	0.572
SFA (% of milk fat)	68.1 ± 4.64	66.9 ± 5.68	0.189
MUFA (% of milk fat)	23.3 ± 2.75	24.1 ± 1.98	0.317
PUFA (% of milk fat)	4.08 ± 0.26	4.35 ± 0.16	0.032
Other lipids (% of milk fat)	4.35 ± 0.56	4.82 ± 0.47	0.089

Milk, common milk supplement treatment; KPM, Kelp powder treated milk supplement treatment. CFU, colony forming unit; SCC, somatic cell counts; SFA, saturated fatty acid; MUFA, monounsaturated fatty acid; PUFA, polyunsaturated fatty acids.

### Animals and experimental design

A total of 48 3-week old Kunming (KM) male mice with an average body weight of 16.1 g ± 0.2 g were acquired from Hunan SJA Laboratory Animal Co., Ltd (Changsha, Hunan province, China) http://www.hnsja.com/index.html. All mice were randomly divided into the control treatment (CON, feed with standard chow), the common milk supplement treatment (Milk), and the kelp powder treated milk (KPM). Ingredients and the nutritional levels of the standard chow are listed in Table 2. Each treatment contained eight repeats, with two mice in each repeat. All animals were housed in stand cages (*n* = 2 mice per cage) in the same room, exposed to the environment with 22 ± 2.0°C of temperature, 45 ± 5.0% of humidity, and 12 h light/dark cycle lighting program. Mice were acclimatized for 1 week before the 28-days-long main experiment commenced, and given free access to standard chow and water.

**Table 2:** Ingredients and chemical composition of the basal diet (dry matter basis).

**Items**	**Content (%)**
Ground corn	39.00
Wheat bran	20.00
Wheat flour	15.00
Soybean meal	16.00
Soybean oil	1.00
Fish meal	4.00
NaCl	0.50
Limestone	1.50
CaHPO_4_	2.00
Premix^a^	1.00
**Nutritional levels (%)**	
GE (MJ/kg)	14.21
EE	4.50
CP	18.10
Ash	4.00
Lys	0.82
Met+Cys	0.53
Ca	1.20
P	0.80

a The components contained in the premix are as following: Fe, 100 mg; Cu, 12 mg; Mn, 75 mg; Zn, 30 mg; Se, 0.1 mg; I, 0.5 mg; VA, 900,000 IU; VB_1_, 8 mg; VB_2_, 10 mg; VB_6_, 6 mg; VD, 800 IU; VE, 60 IU.

### Parameters measurement

#### Growth performance

Body weight gain (BWG) and feed intake (FI) were calculated through the discrepancy between the initial supply and the end residue and further displayed as the average of replicates. Feed conversion ratio (FCR) was calculated by the following equation of FI/BWG.

### Serum lipids-related parameters and antioxidant capacity measurement

At the end of the study, one mouse of each repeat was selected for blood collection through the orbital venous plexus method (17). Serum was separated through coagulation at room temperature for 30 min and centrifuged at 3,000 *g* for 10 min. All samples were then stored at −20°C until the analysis.

Serum lipids-related parameters included cholesterol, triglyceride (TG), high-density lipoprotein (HDL), and low-density lipoprotein (LDL), which were determined by kits detection methods. All the assay kits were acquired from the Nanjing Jiancheng Biotech Company (Nanjing, Jiangsu Province, China http://www.njjcbio.com). All measurements were operated through the AU5421 Automatic Biochemistry Analyzer (BackmanKelt, USA) at the First Affiliated Hospital of Nanchang University.

Further, blood antioxidant parameters which included Superoxide Dismutase (SOD), glutathione peroxidase (GSH-px), and malondialdehyde (MDA) were measured by kits detection methods to determine the effects of KPM supplementation on the antioxidant capacity of mice. The kits used were also acquired from the Nanjing Jiancheng Biotech Company and operated through the AU5421 Automatic Biochemistry Analyzer (BackmanKelt, USA) at the First Affiliated Hospital of Nanchang University.

### Activities of liver lipometabolic-related enzymes measurement

The mice that were used for blood collection were selected and fasted for 16 h, anesthetized, and humanely sacrificed. Liver samples of each mouse were then acquired and rapidly frozen with liquid nitrogen and stored at −80°C for further enzymatic activity analysis.

Activities of fatty acids synthetase (FAS), acetyl-CoA carboxylase (ACC), lipoprotein esterase (LPL), adipose triglyceride lipase (ATGL), glycerol-3-phosphate acyltransferases (GPAT), and carnitine palmitoyltransferase 1 (CPT1) were determined through the ELISA method which was detailed in our previous study (18). Simply stated, all kits used were acquired from the Nanjing Jiancheng Bioengineering Co., Ltd. (Nanjing, Jiangsu Province, China, http://www.njjcbio.com). To be simply stated, 0.1 g of liver tissue was separated according to the manufacturer's instructions for each enzyme measurement procedure. Liver tissue was mixed with the tissue diluent (five times the volume of the tissue) into homogenate and then centrifuged at 4°C with 3,000 rpm for about 30 min, followed by an interactive reaction with coloration liquid on ice for the chromogenic reaction for about 20 min after the supernatant was separated. UV spectrophotometer (Agilent Cary 60) was used for detecting the FAS, ACC, LPL, ATGL, GPAT, and CPT1 absorbance (19).

The activity of each enzyme was calculated using the following curve:


$$\begin{array}{l} Activity_{\text{n}}\left(U/g\right)=\left[\left(\Delta A_{\text{R}}-\Delta A_{\text{B}}\right)\times V_{\text{t}}/\varepsilon_{\text{n}}/d\times 10^{6}\right]/V_{\text{S}}/T \end{array}$$

where, ε_n_ = molar extinction coefficient of the enzyme, d = optical path of the cuvette, V_t_ = total mixed volume, V_S_ = sample volume, T = reaction time, ΔA_R_ = absorbency of reaction cuvette, and ΔA_B_ = absorbency of the blank.

### Gut development

Ileum and jejunum samples of each mouse were collected for the paraffin section. Ten villuses of each intestinal segment were chosen for measuring the villus height and crypt depth in a random order to avoid bias. The ratio of villus height to crypt depth was then calculated to see the development of the intestinal wall.

### Cecal microbiota

Cecal samples collected at the end of the trial after mice were sacrificed, and samples were rapidly frozen with liquid nitrogen and stored at −80°C for further 16S rRNA sequencing analysis.

The sequencing method of 16S rRNA bacterial diversity analysis has been well-stated in He et al. (20). In brief, DNA from each sample was extracted using the CTAB/SDS method followed by the measurement of DNA concentration and purity. The V4 region of the 16S rRNA gene was amplified using the universal primers 520F and 802R (F: GTGCCAGCMGCCGCGGTAA and R: GGACTACHVGGGTWTCTAAT) for the PCR amplification process. The mixture of PCR products was purified with Qiagen Gel Extraction Kit (Qiagen, Hilden, Germany). Sequencing libraries were established using TruSeq^®^ DNA PCR-Free Sample Preparation Kit (Illumina, USA) followed by an assessment of the library quality using the Qubit@ 2.0 Fluorometer (Thermo Scientific) and Agilent Bioanalyzer 2100 system, and a sequencing process Illumina HiSeq 4000 platform (Illumina Inc., San Diego, USA). Quantitative Insights Into Microbial Ecology (QIIME, V1.7.0) quality controlling process were subsequently applied to the quality of the raw tags filtering process to obtain high-quality clean tags. Sequences with similarity >97% were assigned to the same operational taxonomic unit (OTU). GreenGene Database was used based on the SILVA classifier algorithm to annotate taxonomic information.

### Statistical analysis

A normal distribution test on growth performances and serum lipids-related parameters analysis was first conducted using the SAS procedure “proc univariate data = test normal” followed by a one-way ANOVA S-N-K test to investigate the differences. Results were presented as means ± SEM. Relative abundances of cecal bacteria were first received a normal distribution test and further received differential analysis using one-way ANOVA S-N-K test of SAS 9.2 (SAS Institute Inc., Cary, NC, USA). *P*-value < 0.05 was considered to be significant and 0.05 ≤ *P* < 0.10 was considered as a tendency. Principal coordinate analysis (PCoA) for different rumen bacteria was conducted using R package version 3.3.1 (R Core Team, Vienna, Austria). Spearman correlation analysis between the bacteria communities and phenotypic parameters was assessed using the PROC CORR procedure of SAS 9.2. Meanwhile, the correlation analysis between the phenotypic parameters and different milk compositions conducted the same calculation. Then the correlation matrix was created and visualized in a heatmap format using R package version 3.3.1.

## Results

### Growth performances

Growth performances included body weight gain (BWG), feed intake, and feed conversion ratio (FCR) were first measured and the results are shown in Table 3. Average daily feed intake (ADFI) was measured in two ways: one directly calculated the deviation between offered and residue, while the other further added to the dry matter content of milk supplement into the deviation. Finally, body weight and total BWG were significantly increased after milk and KPM supplement (*P* < 0.05). Meanwhile, no significant changes were found for both feed intakes calculated with or without milk addition (*P* > 0.05), which indicated a significantly lower FCR after Milk and KPM supplements compared with CON (*P* < 0.05). No significant discrepancies were detected between Milk and KPM treatments for all growth performances (*P* > 0.05). However, KPM showed better BWG and FCR performances.

**Table 3:** Effects of Milk and KPM supplement on the growth performances of KM mice.

**Items**	**CON**	**Milk**	**KPM**	**SEM**	***P*-value**
Initial BW (g)	16.14	16.13	16.09	0.18	0.637
Final BW (g)	37.73^b^	38.58^a^	39.14^a^	0.62	0.034
BWG (g)	21.59^b^	22.45^a^	23.05^a^	0.53	0.041
ADFI^c^ (g)	4.48	4.34	4.39	0.11	0.098
ADFI^d^ (g)	4.48	4.47	4.52	0.07	0.134
FCR	7.26^a^	6.96^b^	6.86^b^	0.13	0.029

a, b Means a significant difference was detected in the same row among treatments. CON, control treatment; Milk, common milk supplement treatment; KPM, Kelp powder treated milk supplement treatment; SEM, standard error of the mean. BW, body weight; BWG, body weight gain;

**ADFI^c^:** , average daily feed intake of basal diet.

**ADFI^d^:** , average daily feed intake of basal diet together with milk intake (dry matter basis); FCR, feed conversion ratio.

### Serum lipids content and antioxidant capacity

Serum contents related to lipid metabolism which included cholesterol, triglyceride (TG), high-density lipoprotein (HDL), and low-density lipoprotein (LDL) are detected and shown in Table 4. Blood cholesterol, TG, HDL, and LDL showed no significant discrepancies after both common milk and KPM supplementation compared with CON (*P* > 0.05). However, the KPM supplement decreased the serum LDL, while it increased the HDL contents, although not significantly.

**Table 4:** Effects of Milk and KPM supplement on the blood lipid-metabolism related parameters of KM mice.

**Items (mmol/L)**	**CON**	**Milk**	**KPM**	**SEM**	***P*-value**
Total cholesterol (TC)	1.78	1.72	1.56	0.167	0.131
Triglyceride (TG)	0.33	0.32	0.28	0.028	0.095
HDL	1.33	1.31	1.36	0.049	0.182
LDL	0.30	0.31	0.28	0.020	0.313

CON, control treatment; Milk, common milk supplement treatment; KPM, Kelp powder treated milk supplement treatment; SEM, standard error of the mean. HDL, high-density lipoprotein; LDL, low-density lipoprotein.

**Figure 1:**
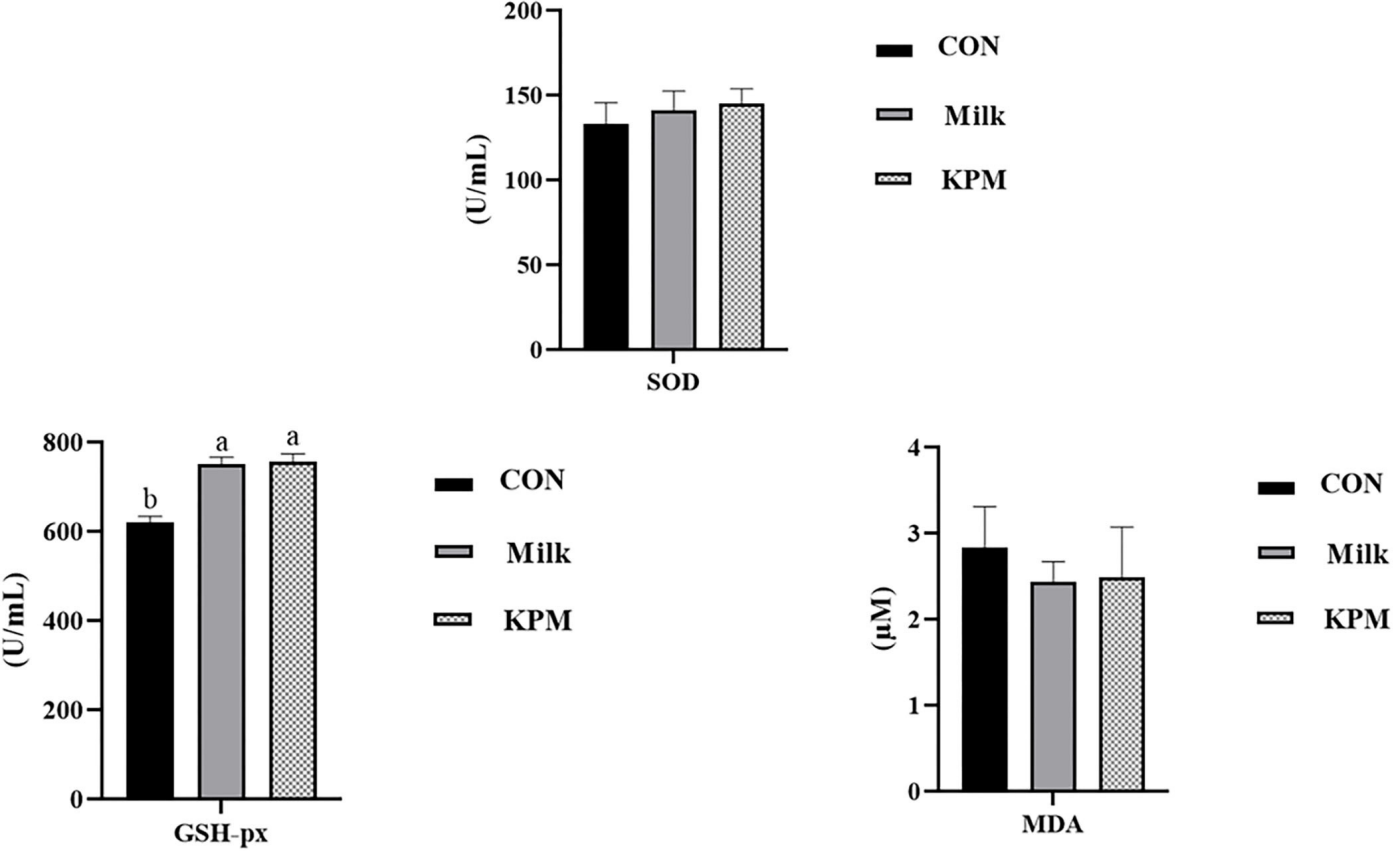
Serum antioxidant capacity evaluation of CON, Milk, and KPM treatments. GSH-PX, glutathione peroxidase; SOD, superoxide dismutase; MDA, malondialdehyde; CON, control treatment; Milk, common milk supplement treatment; KPM, Kelp powder treated milk supplement treatment. Different letters mean a significant difference was detected among treatments.

Further, serum antioxidant parameters were measured and the results are shown in Figure 1. Common milk and KPM supplements significantly increased the content of GSH-px compared with CON (*P* < 0.05). Besides, KPM effectively increased the serum SOD content while decreasing the MDA content compared with CON, however, not significantly. No significant discrepancy was observed between the Milk and KPM treatments.

### Liver lipometabolic-related enzymes

**Figure 2:**
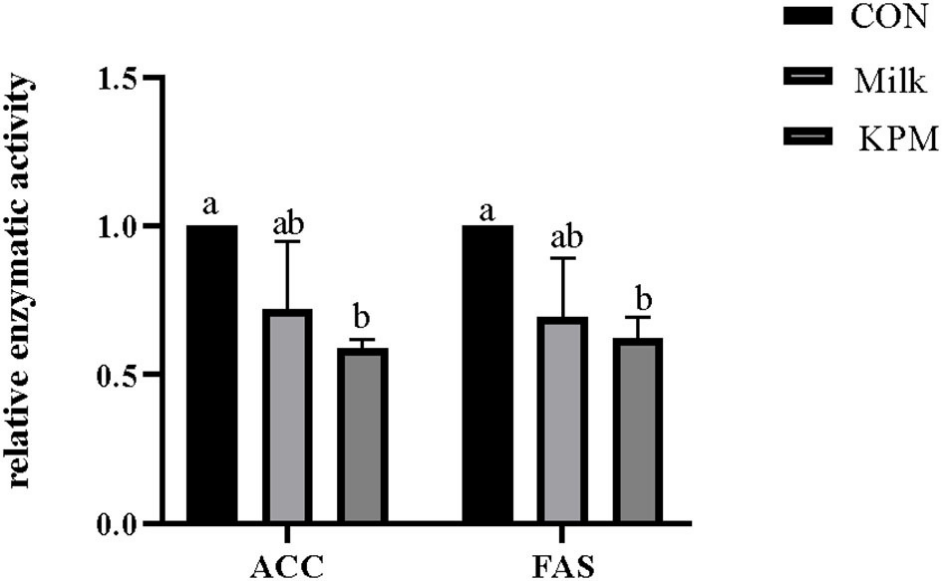
Effects of Milk and KPM supplement on the enzymatic activity of ACC and FAS of KM mice. FAS, fatty acids synthetase; ACC, acetyl-CoA carboxylase; CON, control treatment; Milk, common milk supplement treatment; KPM, Kelp powder treated milk supplement treatment. Different letters mean a significant difference was detected among treatments.

**Figure 3:**
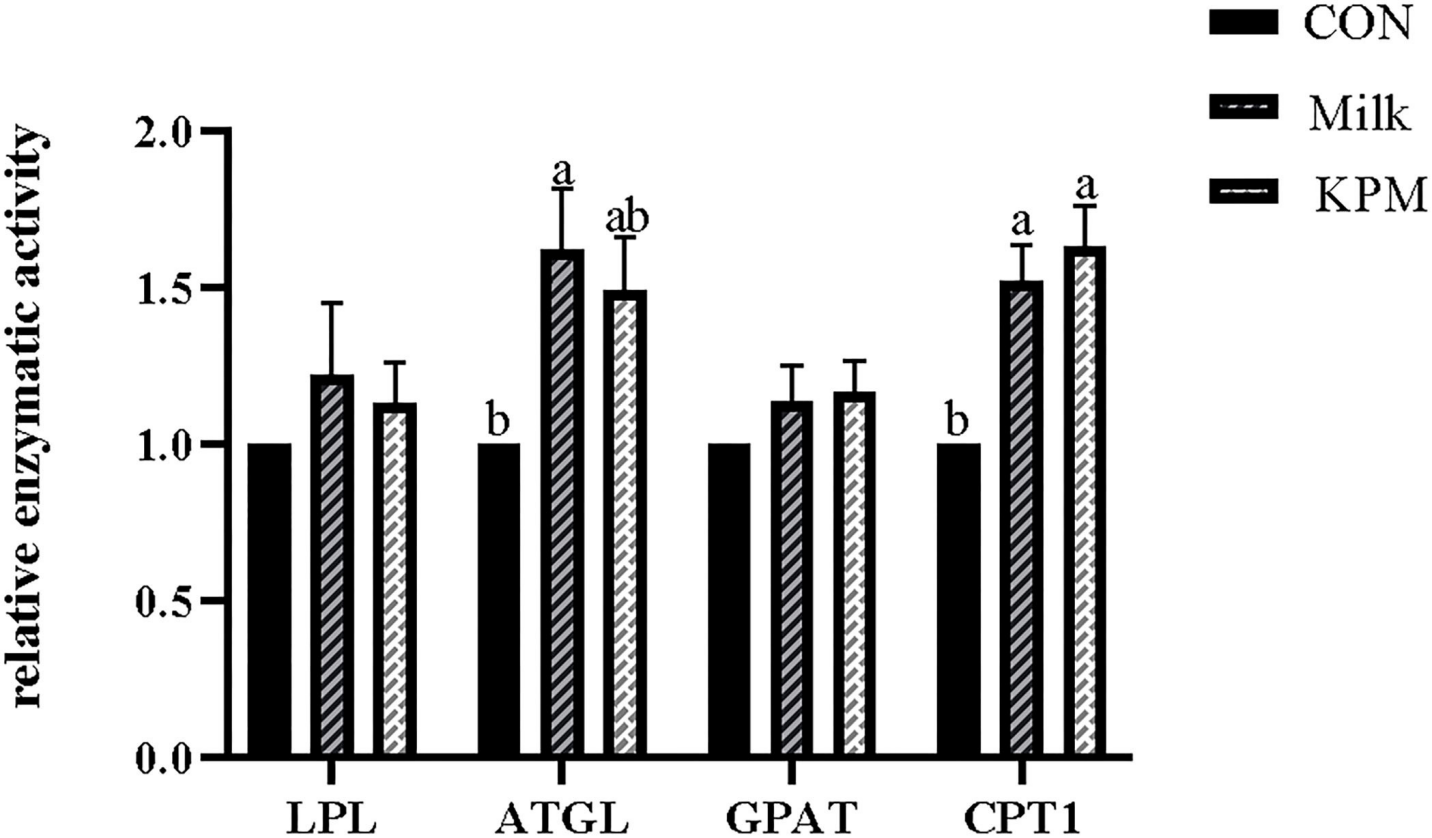
Effects of Milk and KPM supplement on the enzymatic activity of LPL, ATGL, GPAT, and CPT1 of KM mice. LPL, lipoproteinesterase, ATGL, adipose triglyceride lipase; GPAT, glycerol-3-phosphate acyltransferases; CPT1, carnitine palmitoyltransferase 1. Different letters mean a significant difference was detected among treatments.

The liver lipid metabolism-related enzymes included fatty acids synthetase (FAS), acetyl-CoA carboxylase (ACC), lipoprotein esterase (LPL), adipose triglyceride lipase (ATGL), glycerol-3-phosphate acyltransferases (GPAT), and carnitine palmitoyltransferase 1 (CPT1) were selected to investigate the impacts of milk and KPM supplement of physiological lipid metabolism. Results are shown in Figures 2, 3.

Lipogenesis-related enzymatic activities including FAS and ACC were first investigated and shown in Figure 2. Milk and KPM supplements treated effectively decreased the activities of both FAS and ACC compared with CON (*P* < 0.05). No significant differences were found between Milk and KPM treatments (*P* > 0.05).

Lipolysis-related enzyme activities such as LPL, ATGL, GPAT, and CPT1 were subsequently detected and shown in Figure 3. Common milk and KPM supplementation significantly increased the activities of CPT1 compared with CON treatment (*P* < 0.05). Besides, Milk treatment significantly increased the expression of ATGL compared with CON (*P* < 0.05). No significant discrepancies were observed in LPL and GPAT among all the treatments (*P* > 0.05).

### Gut development

**Figure 4:**
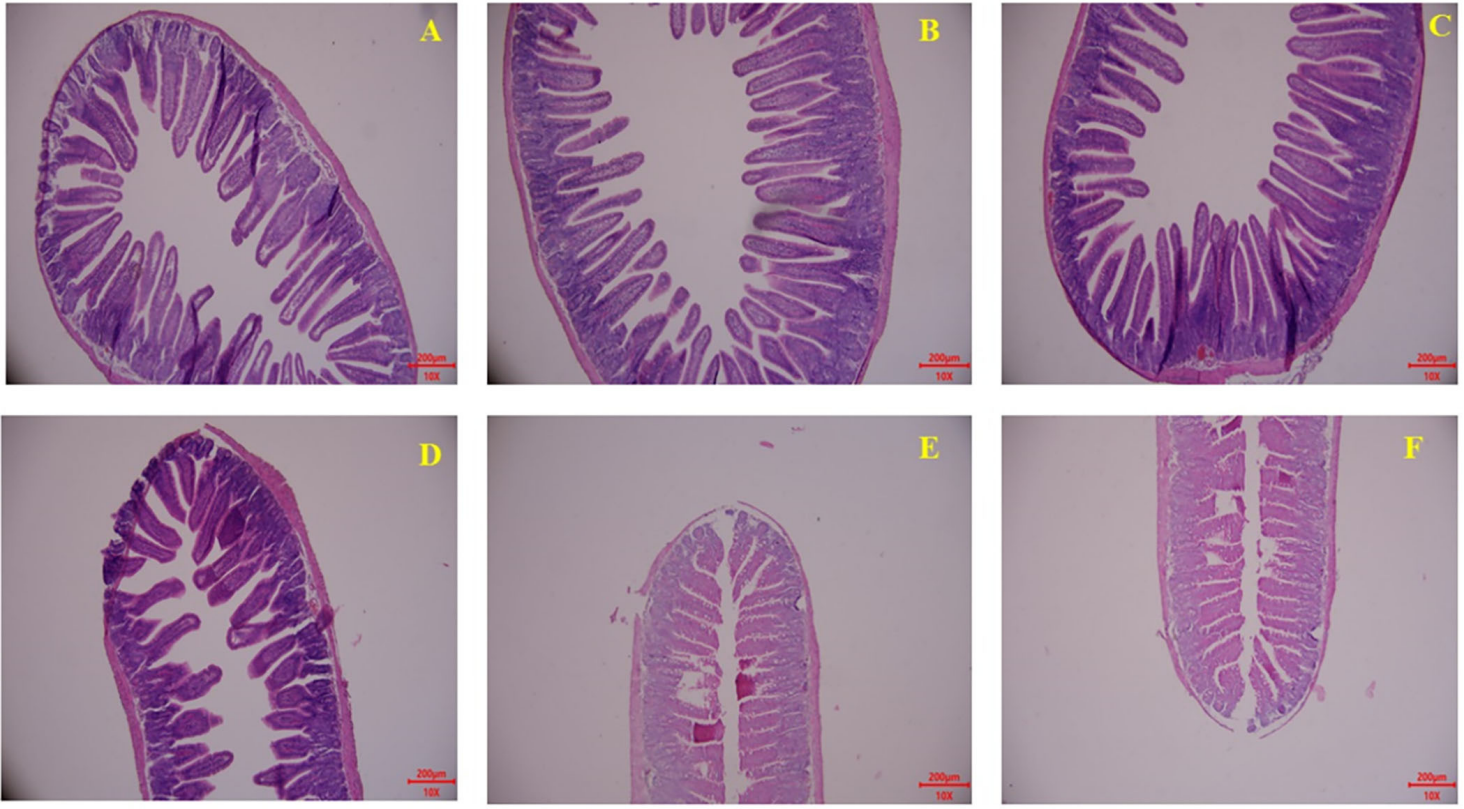
Effects of Milk and KPM supplement on the jejunum and ileum development of KM mice. **(A–C)** Represented the jejunum while **(D–F)** represented the ileum **(A,D)** control treatment; **(B,E)** common milk supplement treatment; **(C,F)** Kelp powder treated milk supplement treatment.

Gut development (mainly including jejunum and ileum) related parameters including the villus height and crypt depth were measured through gut development paraffin section results are displayed in Figure 4 and Table 5. Milk and KPM treatments showed significant promoting effects on jejunum villus development and resulted in a significantly higher ratio of V/C (*P* < 0.05) compared with CON. However, no enhancement effects were found for ileum development (*P* > 0.05). No significant differences were found between Milk and KPM treatments for both jejunum and ileum development (*P* > 0.05).

**Table 5:** Effects of Milk and KPM supplement on the gut development-related parameters of KM mice.

	**Items**	**CON**	**Milk**	**KPM**	**SEM**	***P*-value**
Jejunum	Villus height	397.1^b^	412.6^a^	416.3^a^	9.73	0.017
	Crypt depth	62.42	63.82	62.81	2.56	0.347
	V/C	6.36^b^	6.47^b^	6.63^a^	0.13	0.046
Ileum	Villus height	236.9	228.7	233.6	10.71	0.187
	Crypt depth	48.06	47.18	48.35	1.95	0.274
	V/C	4.93	4.85	4.83	0.24	0.623

a, b Means a significant difference was detected in the same row among treatments.

CON, control treatment; Milk, common milk supplement treatment; KPM, Kelp powder treated milk supplement treatment; SEM, standard error of the mean.

### Gut microbiota analysis

Gut microbiota was further investigated following the intestinal development analysis. Generally, after quality control, a total of 12 phyla, and more than 200 genera were identified, and these bacteria are shown in Supplementary Table 1. All taxonomy results are selected for further α-diversity and β-diversity analysis.

#### α-diversity

Alpha diversity was first used in analyzing the complexity of bacterial diversity through Chao1, Shannon, Simpson, ACE, and Observed_species indexes. Results in Table 6 showed that significant increases of Chao1 and ACE indexes after KPM supplement were acquired compared with CON (*P* < 0.05), while common milk supplement showed no alteration in cecal bacterial α-diversity compared with CON. No significant alterations were detected on Shannon, Simpson, and Observed_species among all three treatments (*P* > 0.05).

**Table 6:** Effects of Milk and KPM supplement on the α-diversity of KM mice cecal microbiota.

**Items**	**CON**	**Milk**	**KPM**	**SEM**	***P*-value**
Shannon	5.61	5.71	5.70	0.09	0.305
Simpson	0.93	0.94	0.94	0.006	0.174
ACE	842.7^b^	877.1^ab^	912.7^a^	37.7	0.042
Chao1	853.5^b^	875.7^b^	920.6^a^	38.6	0.014
Observed_species	723.7	742.5	752.3	30.6	0.114

a, b Means a significant difference was detected in the same row among treatments.

SEM, standard error of the mean; CON, control treatment; Milk, common milk supplement treatment; KPM, Kelp powder treated milk supplement treatment.

#### β-diversity

**Figure 5:**
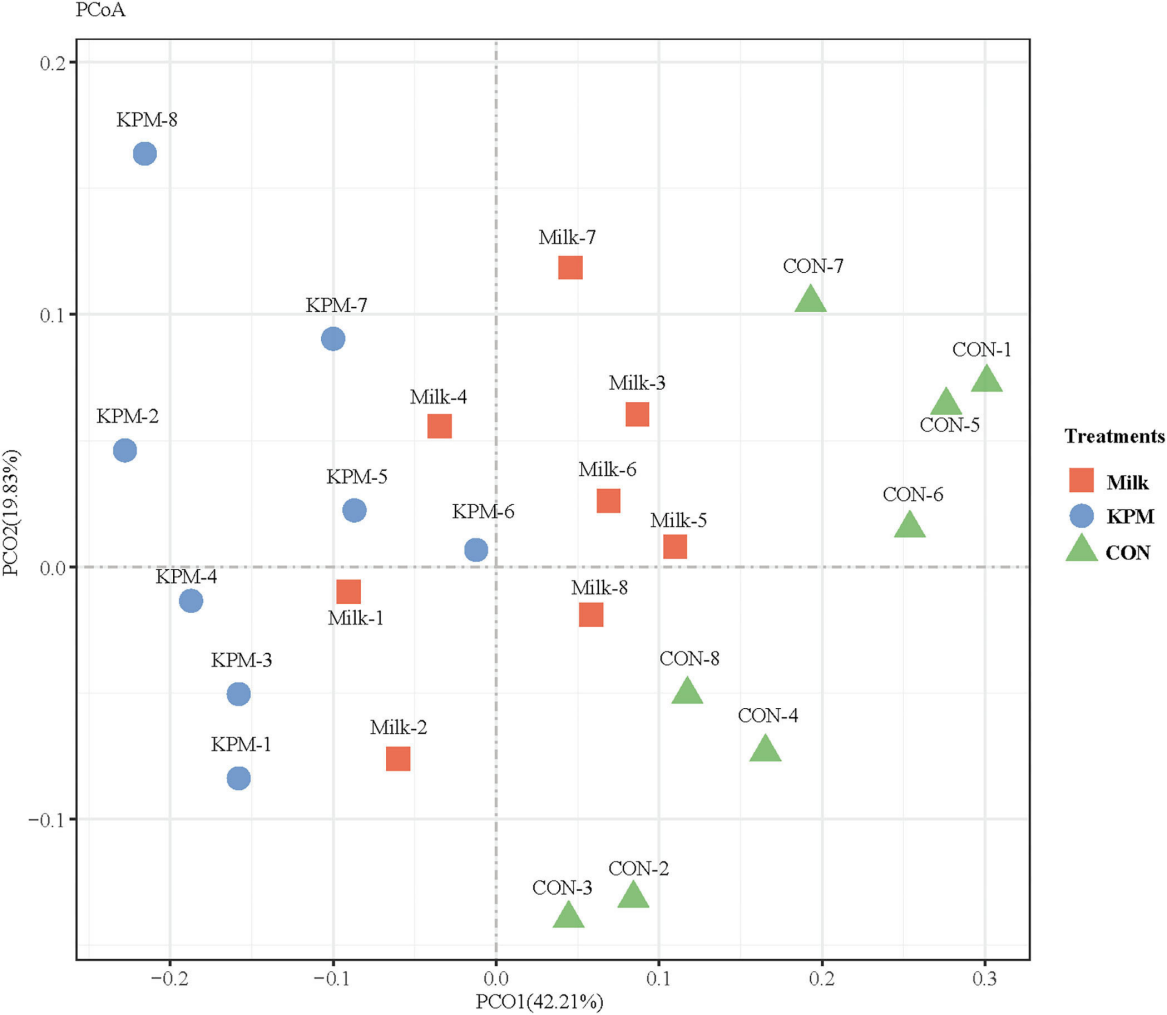
Principal coordinate analysis (PCoA) on community structures of the gut microbiota of CON, Milk, and KPM treatments. CON, control treatment; Milk, common milk supplement treatment; KPM, Kelp powder treated milk supplement treatment.

Principal coordinates analysis (PCoA) which mainly clarified the monolithic discrepancy of bacterial profiles among all treatments proceeded. As shown in Figure 5, PCoA axes 1 and 2 accounted for 42.21 and 19.83%, respectively. Bacterial communities after both Milk and KPM treatments could be separated from those in CON through PCoA axes 1 and 2. Besides, bacterial communities in KPM treatment could be separated from those in Milk treatment as well, except for KPM6.

Differential relative bacterial abundances in both phyla and genera levels were investigated, and all results are shown in Tables 7, 8. *Firmicutes* contributed the largest population of whole bacteria which accounted for 61% in CON, 55% in Milk, and 52.6% in KPM, respectively. *Bacteroidetes* and *Proteobacteria* occupied the 2nd and 3rd of the total microbiota. KPM and Milk supplement treatments significantly promoted the proliferation of *Proteobacteria*, while suppressing *Firmicutes* compared with CON (*P* < 0.05). Relative abundance of *Bacteroidetes* significantly increased in KPM treatment compared with CON (*P* < 0.05); however, no significant change was observed between Milk and CON. KPM treatment remarkably increased the relative abundance of *Proteobacteria* (*P* < 0.05). No other significant changes were found in other phyla among all three treatments.

**Table 7:** Effects of Milk and KPM supplement on the relative abundances of KM mice cecal microbiota in the level of phyla (*n* = 8).

**Phyla (%)**	**CON**	**Milk**	**KPM**	**SEM**	***P*-value**
*Firmicutes*	61.06^a^	55.21^b^	52.66^b^	3.05	0.017
*Bacteroidetes*	33.18^b^	34.43^ab^	35.75^a^	1.14	0.041
*Proteobacteria*	4.15^c^	6.53^b^	10.48^a^	1.13	0.001
*Tenericutes*	0.33	0.24	0.23	0.16	0.253
*Actinobacteria*	0.38	0.32	0.62	0.07	0.222
*Elusimicrobia*	0.16	0.18	0.16	0.12	0.478
*Synergistetes*	0.29	0.27	0.51	0.05	0.098
*Verrucomicrobia*	0.11	0.09	0.12	0.02	0.171
Others	0.34	0.72	0.47	006	0.214

a, b, c Means a significant difference was detected in the same row among treatments.

SEM, standard error of the mean; CON, control treatment; Milk, common milk supplement treatment; KPM, Kelp powder treated milk supplement treatment.

**Table 8:** Effects of Milk and KPM supplement on the relative abundances of KM mice cecal microbiota in the level of genera (*n* = 8).

**Genera (%)**	**CON**	**Milk**	**KPM**	**SEM**	***P*-value**
*Faecalibacterium*	16.23^a^	14.12^b^	13.17^b^	1.17	0.017
*Parabacteroides*	10.67^b^	13.49^a^	14.01^a^	2.14	0.041
*Ruminococcaceae*	12.21^a^	9.01^b^	10.06^b^	1.13	0.031
*Prevotella*	10.77^b^	13.77^a^	14.11^a^	1.06	0.023
*Alistipes*	9.98	8.41	8.58	0.97	0.222
*Ruminococcus*	9.38	10.64	10.03	1.12	0.478
*Oscillospira*	6.08	4.94	6.62	1.05	0.098
*Megamonas*	2.86	0.58	1.01	0.92	0.071
*Sutterella*	2.59	4.18	3.65	0.96	0.214
*Phascolarctobacterium*	2.54^b^	7.04^a^	4.11^b^	1.76	0.031
*Helicobacter*	1.26	4.18	1.34	1.22	0.478
*Butyricimonas*	1.47	1.02	1.20	0.85	0.398
*Coprococcus*	1.30	0.99	1.28	0.59	0.171
*Lactobacillus*	0.38^b^	0.50^a^	0.56^a^	0.16	0.014
*Methanobrevibacter*	0.27	0.10	0.14	0.07	0.064
*Bifidobacterium*	0.13^b^	0.17^a^	0.19^a^	0.02	0.021
*Clostridium*	0.11^b^	0.19^a^	0.21^a^	0.04	0.033
*Campylobacter*	0.15^b^	0.09^b^	0.31^a^	0.07	0.013
*Streptococcus*	0.07^a^	0.02^b^	0.04^b^	0.01	0.008
Others	9.73	9.37	9.42	0.48	0.323

a, b Means a significant difference was detected in the same row among treatments.

SEM, standard error of the mean; CON, control treatment; Milk, common milk supplement treatment; KPM, Kelp powder treated milk supplement treatment.

When referred to genera, *Faecalibacterium, Parabacteroides, Prevotella*, and *Ruminococcaceae* accounted for the top four genera of all three treatments. Compared with CON, *Faecalibacterium, Ruminococcaceae*, and *Streptococcus* are significantly decreased (*P* < 0.05), while *Parabacteroides, Prevotella, Lactobacillus, Clostridium*, and *Bifidobacterium* are significantly increased (*P* < 0.05) after both common milk and KPM supplement treatments. Besides, *Phascolarctobacterium* is significantly proliferated in Milk compared with the other two treatments (*P* < 0.05), and KPM treatment significantly increased the relative abundance of *Campylobacter* (*P* < 0.05). No significant changes were investigated in other genera (*P* > 0.05).

### Correlation analysis between growth performances, blood lipids content, gut development, and cecal microbiota

**Figure 6:**
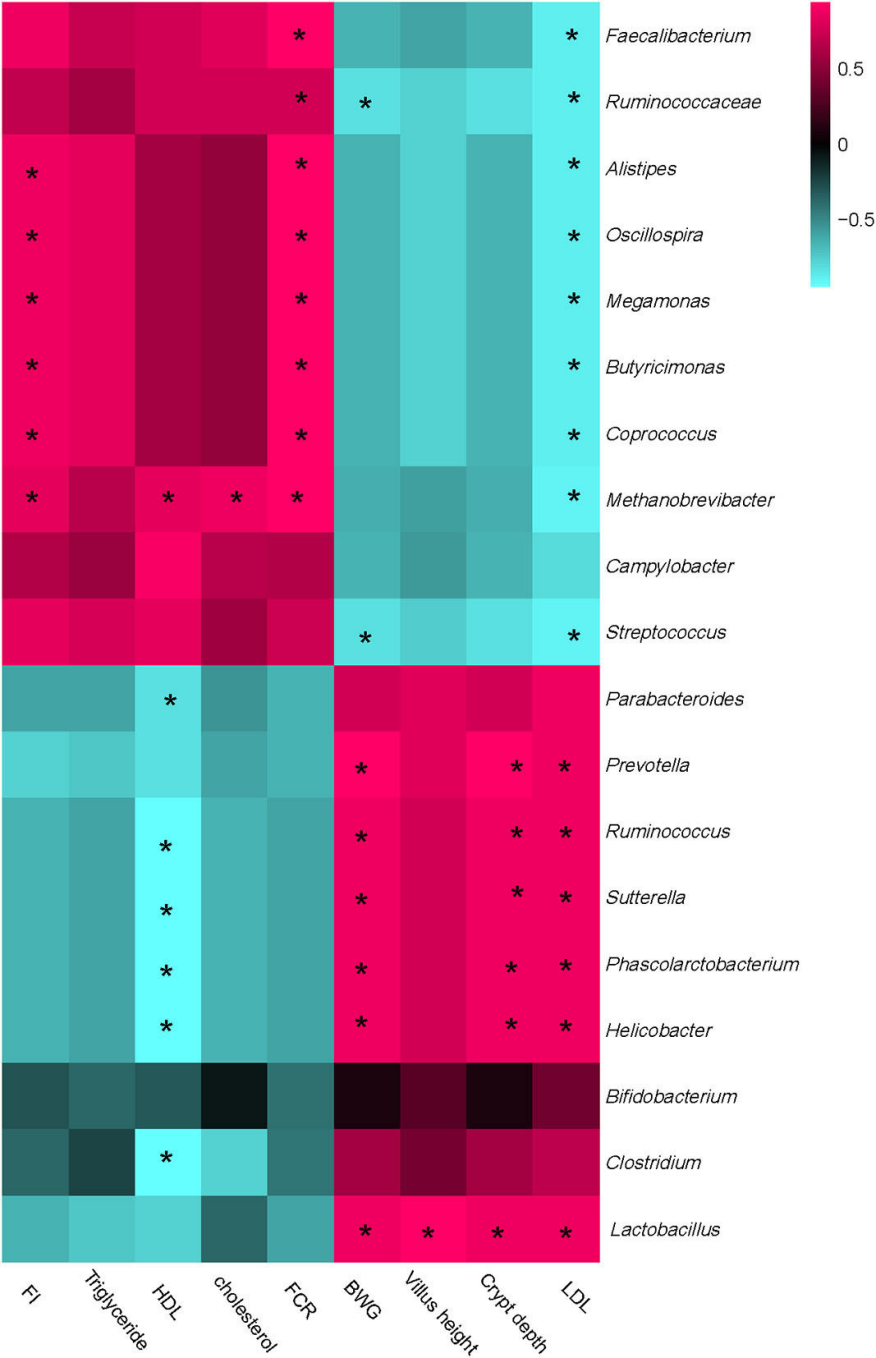
Correlation analysis between abundances of cecal bacteria and mice production performances, intestinal development parameters, and blood lipids parameters on the level of genera. The red color represents a positive correlation while the blue color represents a negative correlation. “*” Means a significant correlation (|r| > 0.55, *P* < 0.05). FI, feed intake; BWG, body weight gain; FCR, feed conversion ratio; HDL, high-density lipoprotein; LDL, low-density lipoprotein.

Interactive analysis between the most abundant bacteria and production performance, carcass performance, and intestinal development parameters were conducted and the result is shown in Figure 6.

Integrally, all parameters could be separated into two big clusters. One mainly consisted of FI, HDL, triglyceride, cholesterol, and FCR, which showed a positive correction with *Faecalibacterium, Ruminococcaceae, Alistipes, Oscillospira, Megamonas, Coprococcus, Methanobrevibacter, and Streptococcus*, while negatively correlated with *Prevotella, Parabacterioides, Ruminococcus, Lactobacillus*, and *Bifidobacterium*. The other cluster mainly consisted of LDL, Villus height, Crypt depth, and BWG, which showed a complete converse correlation with the cecal microbiota compared with the former one.

Particularly, FI and FCR were strongly positively correlated with *Alistipes, Oscillospira, Megamonas, Coprococcus, and Methanobrevibacter*, while the BWG showed a positive relation with *Prevotella, Parabacterioides, Ruminococcus, Lactobacillus, and Helicobacter*. HDL was strongly negatively correlated with *Prevotella, Parabacterioides, Ruminococcus, Helicobacter, and Clostridium*, while the LDL showed a complete correlation compared with HDL. Besides, LDL was significantly negatively correlated with *Faecalibacterium, Ruminococcaceae, Alistipes, Oscillospira, Megamonas, Coprococcus, Methanobrevibacter*, and *Streptococcus*. No other significant correlations were acquired between cecal microbiota and phenotypic parameters.

### Relationship between growth performances, gut development, serum antioxidant capacity, and different milk composition parameters

**Figure 7:**
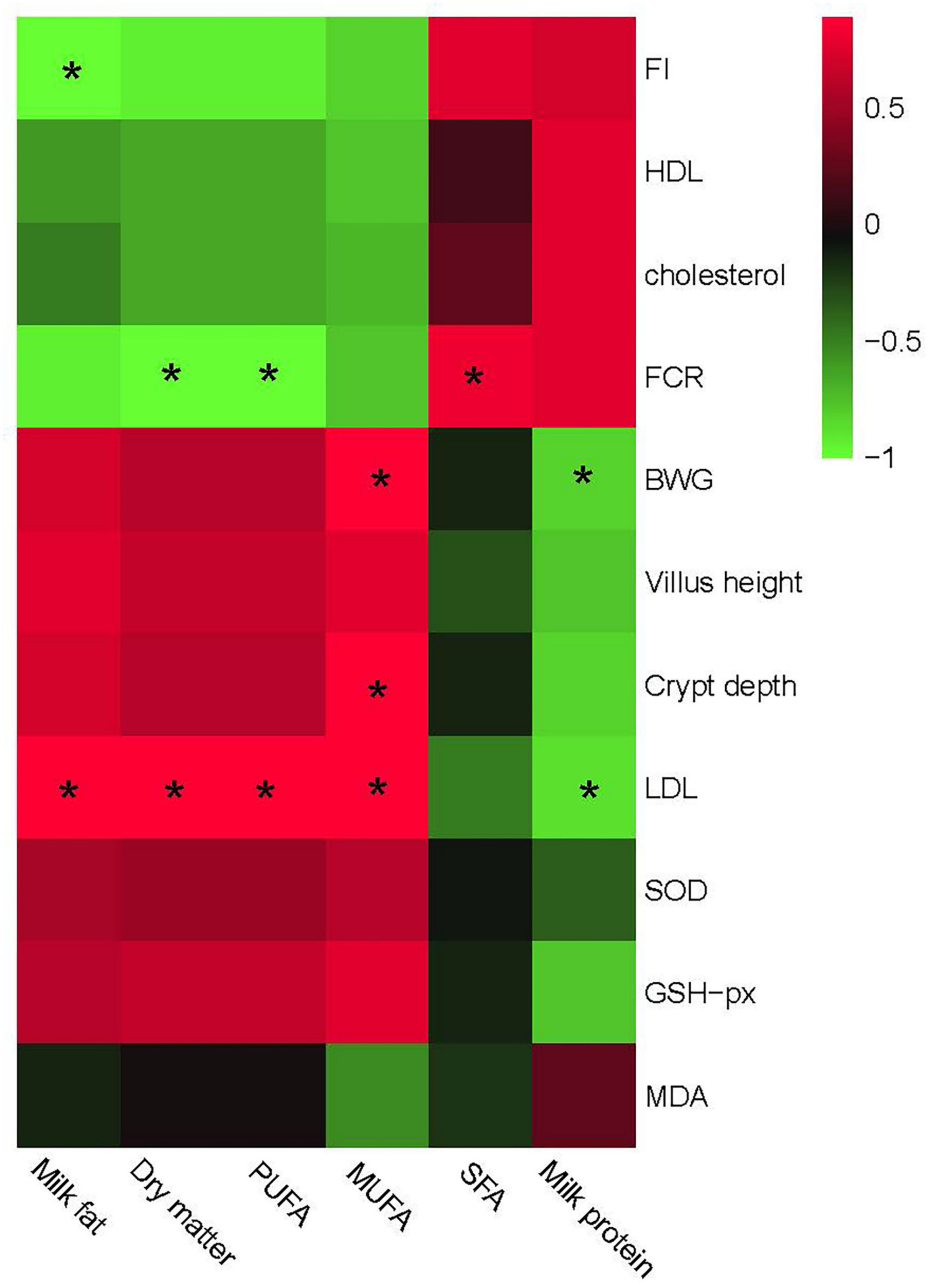
Relationship between growth and gut development, serum antioxidant capacity, and different milk composition parameters. The red color represents a positive correlation while the green color represents a negative correlation. “*” Means a significant correlation (|r| > 0.55, *P* < 0.05). FI, feed intake; BWG, body weight gain, FCR, feed conversion ratio; HDL, high-density lipoprotein; LDL, low-density lipoprotein; SFA, saturated fatty acid; MUFA, monounsaturated fatty acid; PUFA, polyunsaturated fatty acids.

Finally, we investigated the effects of altered milk composition on the health-related indexes such as growth performances, gut development, and serum antioxidant capacity. The results are shown in Figure 7.

Similar to the correlation analysis between bacteria and phenotypic performances, milk composition could be separated into two big clusters based on the relationship with phenotypic performances. One mainly consisted of milk fat, PUFA, and MUFA, which showed a positive correlation with BWG, gut development including villus height, and serum antioxidant capacities including SOD and GSH-px, and a negative correlation with FCR, feed intake, cholesterol, and HDL contents. The other part mainly consisted of SFA and milk protein, which showed a complete converse correlation with the former one. Particularly, PUFA in this research showed a positive correlation with LDL, while a negative correlation with FCR. MUFA was positively correlated with LDL, crypt depth, and BWG. No other significant correlations were observed between milk composition and phenotypic parameters.

## Discussion

Milk nowadays becomes a necessity in people's daily life, and provides the essential nutrients for body growth (21). The milk content of bioactive ingredients also benefits human (especially infants) health. Therefore, an increment of milk bioactive ingredient content could ulteriorly promote the milk's nutritional value and benefit peoples' life. In our present study, the PUFAs-increased milk was used in analyzing its effects on growth performances and lipid metabolism of mice, purposely to determine the underlying mechanism of KPM on the follow-up application in human daily life.

### Effects of kelp powder-treated milk on body health and growth performances

Being frequently used as a dietary additive in promoting PUFAs content, milk PUFAs content could markedly increase with the proper amount of kelp powder supplement just as our previous study indicated (16). Considering the indiscriminate feed intake of mice in all treatments, the considerable increment of growth performance after KPM treatment might attribute to the incremental intake of PUFAs. Underlying reasons are displayed in the following three aspects.

First of all, increased ingestion of PUFAs help enhance body health. PUFAs were substantiated by hyper-bioactivities in the physiological metabolism process (22), and functionally participated in inflammation alleviation programs other than just being an energy source for body growth (23, 24). Besides, physiological antioxidant capacity was also promoted by a proper increment of PUFAs content, which was reflected by increased serum content of antioxidant substrates, such as GSH-px. The promoted body antioxidant and anti-inflammation capacities may further provide a homeostasis environment for body development and, therefore, induced the increment of body weight.

Besides, the increased body antioxidant capacity exerted positive effects on intestinal barrier functions (25), and thus enhanced gastrointestinal development. Both common milk and KPM contained a certain amount of short-chain fatty acids, especially butyrate, which were well-proved critically promoted intestinal development (9). Long-term utilization of butyrate after milk and KPM supplement helps development. Further, a health-developed gastrointestinal tract, especially the significant increase of villus height improved the contact area between feed and digestive enzymes, which subsequently increased absorption of nutrition ingredients and therefore promoted growth development.

Moreover, a health-developed gastrointestinal tract provided a better habitat environment for bacterial proliferation, which contributed to better growth performance. The gut inhabited trillions of microbiota, synergistically acted on nutrients digestion and absorption, and is modulated by diet management. Increased PUFAs of KPM effectively increased the *Bacteroidetes* to *Firmicutes* ratio and proliferated *Actinobacteria* and *Proteobacteria*, while decreasing the growth of *Enterobacteria* (6, 26, 27). An increase of *Bacteroidetes* and *Proteobacteria* may further help provide more SCFAs and amino acids for tissue development and, therefore, enhancing growth performances. Besides, increased PUFAs are partially metabolized by certain probiotics, such as *Bifidobacteria* and *Lactobacilli* in the distal intestine, which further helps the development of intestine, the enhancement of the absorptivity, and finally helps the increment body weight (28–31).

### Effects of kelp powder-treated milk on physiological lipometabolic process

The liver lipid-metabolism-related enzymes were significantly altered after both KPM supplements compared with the control treatment. These findings of lipometabolic alterations after KPM treatment were in line with Jump (32), who indicated a remarkable lipometabolic regulatory effect existed in PUFAs-abundant substrates. Physiological lipid metabolism complicatedly proceeded throughout the whole life and functionally affected somatic functions. PUFAs in lipid metabolism exert considerable beneficial effects by upregulating the expression of genes involved in fatty acid oxidation while downregulating genes encoding proteins involved in lipid synthesis (27, 33, 34). In our study, increased PUFAs intake in KPM-treated mice significantly upregulated lipolysis-related enzymes including ATGL and CPT1, while significantly downregulated enzymes participated in lipid syntheses, such as FAS and ACC. These alterations of lipometabolic enzymes might help interpret the declined serum content of cholesterol and Triglyceride.

Apart from directly impeding the lipid synthetic process, increased PUFAs might interact with gut microbiota that are involved in nutrient degradation and energy generation processes, and further impact the lipid content in blood and tissues, both in mice and humans (35). A PUFA-rich diet generally prevented lipid accumulation because of the preservation of gut microbial diversity, especially the increase of *Bacteroidetes* and certain amounts of probiotics. The ratio of *Firmicutes*-to-*Bacteroidetes* (F/B) has been well-proved positively associated with obesity (36), the increase of *Bacteroidetes* combined with a decrease of *Firmicutes* help reduce the F/B ratio, which finally caused the decrease of lipid synthesis. Moreover, increased cecal probiotics content such as *Bifidobacterium* and *Lactobacilli* significantly increased nutrient digestion, which causatively lead to more small molecule substances absorbed into the physiological process and reduced the lipid accumulation of peripheral tissue (37, 38).

In summary, kelp-treated milk helped increased the milk PUFAs content, further enhancing the abundance of probiotics and modulating physiological lipid metabolism, and finally promoting growth performances.

## Data availability statement

All the raw data used here have been submitted to NCBI SRA database, and the BioSample accession: SAMN29914696.

## Ethics statement

The animal study was reviewed and approved by JXAULL-20220226.

## Author contributions

HB and ZY designed the study. QM, PM, and JZ conducted the experiment. JZ, SW, CZ, ML, and YS contributed to parameter measurement and data analysis. All authors carefully read and are accountable for all aspects of the work, contributed to the article, and approved the submitted version.

## Funding

This study was supported by the Science and Technology Planning Project of Jiangxi Educational Department (GJJ200414), the Latitudinal Project of Jiangxi Agricultural University (2021JXAUHX021), and the Lv Yang Jin Feng Plan of Yangzhou (YZLYJFJH2019YXBS151).

## Conflict of interest

Authors FX, YS, ML, and ZY were employed by Yangxin Yiliyuan Halal Meat Co. Ltd. The remaining authors declare that the research was conducted in the absence of any commercial or financial relationships that could be construed as a potential conflict of interest.

## Publisher's note

All claims expressed in this article are solely those of the authors and do not necessarily represent those of their affiliated organizations, or those of the publisher, the editors and the reviewers. Any product that may be evaluated in this article, or claim that may be made by its manufacturer, is not guaranteed or endorsed by the publisher.

## Supplementary material

Supplementary Table 1 Taxonomy results of the gut microbiome of each sample.
